# Verminous Typhlitis

**Published:** 1898-02

**Authors:** 


Verminous Typhlitis.—This disease, according to Lucet, is caused by the *heterakis dispar* living exclusively in the cæcum. The symptoms are similar to those common to all verminous diseases: diarrhœa, with yellow or brown, fetid feces, emaciation, pale mucous membranes, and difficulty in walking. The diagnosis is established by the presence of the parasites in the feces. At the necropsy, the cæcum is nflamed and filled with fecal matter containing the parasite. The author recommends the following treatment: a mixture of bread-crumbs, boiled rice, milk, garlic, and wormwood-leaves.—*Rev. Vét.*

